# Interannual measures of nutritional stress during a marine heatwave (the Blob) differ between two North Pacific seabird species

**DOI:** 10.1093/conphys/coab090

**Published:** 2021-11-25

**Authors:** Heidi M Tate, Katharine R Studholme, Alice D Domalik, Mark C Drever, L Michael Romero, Brenna M G Gormally, Keith A Hobson, J Mark Hipfner, Glenn T Crossin

**Affiliations:** 1 Department of Biology, Dalhousie University, Halifax, Nova Scotia, Canada; 2 Wildlife Research Division, Environment and Climate Change Canada, Delta, British Columbia, Canada; 3 Centre for Wildlife Ecology, Simon Fraser University, Burnaby, British Columbia, Canada; 4 Department of Biology, Tufts University, Medford, MA USA; 5 Schmid College of Science and Technology, Chapman University, Orange, CA USA; 6 Department of Biology, University of Western Ontario, London, Ontario, Canada; 7 Environment and Climate Change Canada, Saskatoon, Saskatchewan, Canada

**Keywords:** the Blob, stable isotopes, Pacific Decadal Oscillation, nutritional stress, feather corticosterone, Alcidae

## Abstract

‘The Blob’, a mass of anomalously warm water in the Northeast Pacific Ocean peaking from 2014 to 2016, caused a decrease in primary productivity with cascading effects on the marine ecosystem. Among the more obvious manifestations of the event were seabird breeding failures and mass mortality events. Here, we used corticosterone in breast feathers (fCort), grown in the winter period during migration, as an indicator of nutritional stress to investigate the impact of the Blob on two sentinel Pacific auk species (family Alcidae). Feathers were collected from breeding females over 8 years from 2010 to 2017, encompassing the Blob period. Since Pacific auks replace body feathers at sea during the migratory period, measures of fCort provide an accumulated measure of nutritional stress or allostatic load during this time. Changes in diet were also measured using δ^15^N and δ^13^C values from feathers. Relative to years prior to the Blob, the primarily zooplanktivorous Cassin’s auklets (*Ptychoramphus aleuticus*) had elevated fCort in 2014–2017, which correlated with the occurrence of the Blob and a recovery period afterwards, with relatively stable feather isotope values. In contrast, generalist rhinoceros auklets (*Cerorhinca monocerata*) displayed stable fCort values across years and increased δ^15^N values during the Blob. As marine heatwaves increase in intensity and frequency due to climate change, this study provides insight into the variable response of Pacific auks to such phenomena and suggests a means for monitoring population-level responses to climatological variation.

## 1. Introduction

Marine heatwaves have caused major disruptions to ocean ecosystems (Lotze *et al.*, 2019) and are now occurring with increasing frequency and intensity with direct links to global warming (Joh and Di Lorenzo, 2017; Oliver *et al.*, 2018; Smale *et al.*, 2019). In 2013, an enormous mass of unusually warm water, dubbed ‘the Blob’, formed in the Pacific Ocean, moving closer to shore and affecting nearshore environments in late 2013 and peaking in intensity from 2014 to 2016 (Di Lorenzo and Mantua, 2016; Kintisch, 2015). The Blob emerged from the broader Pacific Decadal Oscillation of climate variability characterized by warm and cool periods (Joh and Di Lorenzo, 2017; Laufkötter *et al.*, 2020). However, the Blob was larger in scale and intensity and persisted over a longer timeframe (Joh and Di Lorenzo, 2017; Laufkötter *et al.*, 2020). This major climatic event was associated with reduced delivery of nutrients from the sub-Arctic to lower latitudes, resulting in decreased primary production (Du and Peterson, 2018; Suryan *et al.*, 2021). These impacts at the base of the food web cascaded across the entire ecosystem, ultimately leading to unprecedented die-offs of marine predators at higher trophic levels (Kintisch, 2015).

Seabirds have long been considered ‘sentinels’ of marine environmental conditions (Hazen *et al.*, 2019) as their position atop the marine food web makes them sensitive to environmental changes from bottom-up effects (Velarde *et al.*, 2019). Since the 1980s, extreme warming events have had far-reaching effects on the survival, phenology and breeding success of many seabird species. For example, a strong El Niño in 1997 led to the starvation of thousands of short-tailed shearwaters (*Puffinus tenuirostris*), which washed up emaciated on shorelines of the southern Bering Sea and Gulf of Alaska (Baduini *et al.*, 2001). More recently, the Blob was correlated with breeding failures and mass mortalities of common murres (*Uria aalge*), Cassin’s auklets (*Cerorhinca monocerata*) and red phalaropes (*Phalaropus fulicarius*) in the Northeastern Pacific Ocean (Drever *et al.*, 2018; Jones *et al.*, 2018; Piatt *et al.*, 2020). These die-offs are hypothesized to be a direct result of bottom-up effects driven by reductions in food availability and nutritional quality (Jones *et al.*, 2018; Piatt *et al.*, 2020). In addition to increased mortality, extreme marine climate events may drive reductions in the productivity of surviving individuals (Fairhurst *et al.*, 2017; Sorensen *et al.*, 2009; Sydeman *et al.*, 2006; Williams *et al.*, 2015) because they are left physiologically ill-prepared for breeding (Borstad *et al.*, 2011; Carle *et al.*, 2015; Sorensen *et al.*, 2009).

In this study, we compared the physiological responses to variation in ocean climate between two species of auks (family Alcidae), the Cassin’s auklet and rhinoceros auklet (*C. monocerata)*, breeding on Triangle Island, British Columbia, Canada (Rodway, 1991). We focused on female auks because their primary reproductive traits, particularly egg production and lay date, may be affected in warmer years (Hipfner, 2008; Hipfner *et al.*, 2020a). In both species, we measured corticosterone levels in feathers (fCort) grown in winter during migration (Landys *et al.*, 2006; Wingfield *et al.*, 1998). Corticosterone is the principal hormonal mediator of allostasis or physiological stress in wild birds (Dallman *et al.*, 1993), but when elevated can also play an adaptive role in daily metabolic regulation and energy balance, thus allowing individuals to respond to environmental stochasticity (Wingfield *et al.*, 1998). Corticosterone levels measured in feathers have been used as indicators of physiological and nutritional stress (Romero and Fairhurst, 2016; Will *et al.*, 2015) and as proxies of population-level health (Fairhurst *et al.*, 2017). Recently, increased fCort has been linked to increases in foraging and nutritional stress in rhinoceros auklets (Will *et al.*, 2015). Although seabirds may experience the effects of marine heatwaves year-round, fCort and feather isotope measurements in this study provide insights into pre-breeding conditions of surviving individuals as feathers are grown during the migratory period in February and March, just prior to spring breeding (April in Cassin’s auklets and May in rhinoceros auklets). Variation in fCort can therefore reflect the pre-breeding, oceanic conditions experienced when individuals ranged throughout the Northeast Pacific from California to Alaska (Hipfner *et al.*, 2020b; Studholme *et al.*, 2019).

Using an AIC_c_ model selection framework, we examined how fCort in the two species was affected by annual oceanic conditions and diet, the latter based on feather δ^15^N and δ^13^C values (Hipfner *et al.*, 2014; Sorensen *et al.*, 2009). Feather δ^15^N may reflect increases with trophic levels, while δ^13^C, in addition to smaller trophic effects, also reflects relative use of benthic versus pelagic prey sources (Deniro and Epstein, 1981; Hobson *et al.*, 1994). This study encompassed the temporal entirety of the Blob through its peak in 2014–2016, and after its decline in 2017, as well as preceding cool-water years in 2010, 2011 and 2013, which provide a point of contrast (Di Lorenzo and Mantua, 2016; Kintisch, 2015; Yang *et al.*, 2018). The Blob formed offshore in 2013, outside of the migratory range of both auks, then moved progressively eastwards towards the North American coast near the end of 2013, overlapping with the overwintering range of the birds and remaining there until its decline in late 2016 (Smale *et al.*, 2019; Studholme *et al.*, 2019). Previous studies indicate that zooplanktivorous seabirds, such as the Cassin’s auklets, sometimes exhibit stronger behavioural and demographic responses to climatic variation than more generalist seabirds, such as the rhinoceros auklets (Morrison *et al.*, 2011; Will *et al.*, 2020). This is because when overall ocean biomass decreases, generalist feeders can take prey from multiple species and trophic levels, unlike the Cassin’s auklets that rely on specific copepod species to meet dietary requirements (Kitaysky and Golubova, 2000; Will *et al.*, 2020). Therefore, we predicted greater interannual variation in fCort levels in Cassin’s auklets than in rhinoceros auklets and a peak in fCort at the crest of the Blob around 2015.

## 2. Methods

### 2.1. Data collection

This study took place on Triangle Island, British Columbia, Canada (50° 52′ N, 129° 05′ W), in the traditional territories of the Kwakwaka'wakw indigenous people. The island supports the world’s largest breeding colony of Cassin’s auklets, with more than half a million pairs (Rodway, 1991) and a large rhinoceros auklet breeding colony (Gaston and Dechesne, 2020). Breast feathers were collected primarily in June of 2010, 2011 and 2014–2017 for individual Cassin’s auklets and in June of 2013–2017 for rhinoceros auklets (see Appendix Table A1). Birds were removed from marked breeding burrows, measured and up to eight breast feathers sampled by pulling quickly at the base of the calamus. Peak breast feather replacement in both species occurs in February–March (Ainley *et al.*, 2020; Gaston and Dechesne, 2020; Pyle, 2009), thus corticosterone levels primarily reflect this window of pre-breeding activity at sea (Fairhurst *et al.*, 2017; Landys *et al.*, 2006), although this time frame may be influenced by possible effects of later fCort circulation onto feathers (Aharon-Rotman *et al.*, 2021). The sex of birds was determined using bill depth (Knechtel, 1998; Pyle, 2008), and only feathers from females were used in this study. Field work on the Triangle Island ecological reserve was approved by British Columbia Parks, the Tlatlasikwala First Nations and the Quatsino First Nations (BC Parks: 102237). All wildlife sampling protocols were approved by Simon Fraser University Animal Care Services (2010–2014: 974B-94) and Environment Canada’s Western and Northern Animal Care Committee (2015–2017: 15MH01, 16MH01, 17MH01). Migratory birds scientific permits included BC-10-0017, BC-11-0016, BC-13-0018, BC-14-0026#1, BC-15-0005, BC-16-0012 and BC-17-0028. The banding permit for all years was 10667F.

### 2.1.1. Corticosterone analysis

fCort analyses followed protocols outlined by Lattin *et al.* (2011), using radioimmunoassay for quantification in pg fCort/mm. Of the feathers collected, 4–7 feathers per bird were used for the analysis to standardize sample mass (10 ± 0.2 mg for Cassin’s auklet; 20 ± 0.2 mg for rhinoceros auklet). To reduce variation, samples were processed in three batches: first for Cassin’s auklets only for 2010–2011 and then for each species separately for 2013–2017. In brief, 7 ml of methanol was added to each feather sample. Tubes were sonicated for 30 minutes, then placed in a shaking water bath at 50°C overnight. Feathers were separated using vacuum filtration and methanol was evaporated using nitrogen gas flow. The dried extracts were then reconstituted in 500 μl of Tris–HCl buffer (0.05 M, pH 8). fCort was quantified by radioimmunoassay and samples were run in duplicate. For all assays, the Sigma anti-corticosterone antibody was used (Sigma C8784, St. Louis, MO, USA). For the Cassin’s auklet samples, the mean intra-assay coefficient of variation (CV) was 2.85% and the inter-assay CV was 7.27%. For the rhinoceros auklet samples, the mean intra-assay CV was 3.16% and the inter-assay CV was 14.94%. Different standardized control pools consisting of pulverized European starling feathers were used for the assays as they were completed at different times and therefore we cannot compute an overall inter-assay CV. Samples smaller than 8 mg for Cassin’s auklets (min = 7.3 mg) or 20 mg for rhinoceros auklets (min = 19.1 mg) were retained after finding no evidence of mass bias in our dataset (Cassin’s auklets: F_1,113_ = 0.9376, *P* = 0.335, adjusted R^2^ = −0.005; Rhinoceros auklets: F_1,79_ = 0.0029, *P* = 0.957, adjusted R^2^ = −0.013).

### 2.1.2. Stable isotope analysis

Stable isotope composition was determined using one feather selected at random from each individual. Each feather was soaked in 2:1 chloroform:methanol solution for 24 hours to remove surface oils, rinsed twice with fresh solution and air dried in a fume hood for at least another 24 hours at Dalhousie University. These feathers were then analysed at the Element and Heavy Isotope Analytical Laboratories, University of Windsor Great Lakes Institute for Environmental Research (2010–2011 samples) or Environment and Climate Change Canada stable isotope laboratory in Saskatoon, SK (2014–2017 samples).

At the University of Windsor, feather calami were removed and the remaining feather material was freeze-dried, minced to a fine consistency, subsampled, weighed and combusted in a Costech elemental analyser (Costech International S.P.A., Milan, Italy) interfaced with a Thermo Delta V isotope-ratio mass-spectrometer (Bremen, Germany) to determine δ^15^N and δ^13^C values. For δ^15^N, standard deviation was ±0.10‰ for both internal standard tilapia and NIST standard bovine liver and for δ^13^C, within-run standard deviations were ±0.13‰ for tilapia and ±0.20‰ for bovine liver (Larocque *et al.*, 2021). Additionally, 17 samples were run in duplicate. The two-way intraclass correlation coefficients (‘icc’, R package ‘irr’) for these duplicates were 0.951 for δ^15^N and 0.997 for δ^13^C. Duplicate samples were averaged to yield single values prior to analysis.

At the Environment and Climate Change Canada stable isotope laboratory in Saskatoon, SK, the procedure was similar but calami were removed prior to soaking and samples were not freeze dried. Between 0.5 and 1.0 mg of feather material was combusted online using a Eurovector 3000 elemental analyser (Eurovector, Milan, Italy). The resulting CO_2_ and N_2_ was separated by gas chromatograph and introduced into a Nu Horizon (Nu Instruments, Wrexham, UK; www.nu-ins.com) triple-collector isotope-ratio mass-spectrometer via an open split and compared to CO_2_ or N_2_ reference gas. Using previously calibrated internal laboratory C and N standards [powdered keratin (BWBIII; δ^13^C = −20‰; δ^15^N = 14.4‰) and gelatin (PUGEL; δ^13^C = −13.6‰; δ^15^N = 4.73‰)], within run (*n* = 5), precisions for δ^15^N and δ^13^C measurements were ± 0.15‰.

Results are reported in standard δ notation as parts per thousand (‰) deviation from the international standards Vienna PeeDee Blemenite (VPDB) for δ^13^C and atmospheric air (AIR) for δ^15^N, respectively (see Appendix Table A2). Based on replicate measurements of in-house laboratory standards, measurement precision was estimated to be ±0.2‰ for both isotopes.

**Table 1:** Models predicting lnfCort for female Cassin’s auklets (*P. aleuticus*) and rhinoceros auklets (*C. monocerata*). The global model was lnfCort~ Year + Species + Year*Species. ΔAIC_c_ is the difference between a given model and the top-ranked model, AIC_w_ is the Akaike model weight, model fit provides a measure analogous to R^2^, and is calculated by 1—model deviance/null model deviance where a fit closer to 1 is best. Models with ΔAIC_c_ < 2 are in bold

Parameters	AIC_c_	ΔAIC_c_	AIC_w_	Model fit
**Year + Species**	**15.3**	**0.00**	**0.864**	**0.42**
Species	20.0	4.77	0.080	0.37
Year + Species + Species^*^Year	20.7	5.47	0.056	0.42
Year	77.9	62.63	0.000	0.05
~1 (null model)	78.9	63.67	0.000	0.00

### 2.1.3. Statistical analysis

All analyses were run using R version 4.0.2. fCort was natural log (ln) transformed to best normalize model residuals. Other variables included species, year, feather δ^15^N and feather δ^13^C. Species and year were treated as categorical predictors, while feather isotopes and fCort values were continuous.

We first tested for differences in fCort levels between species using general linear models with the ‘glm’ function, with species and year as predictor variables of lnfCort. Data from 2014 to 2017 were used for these models as these years had data present from both species. The ‘dredge’ function from the MuMIn package was used to generate all combinations of lnfCort ~ Species + Year + Species*Year and to rank each model with Akaike’s Information Criterion corrected for small sample sizes (AIC_c_; Burnham and Anderson, 2004). Only models with ΔAIC_c_ < 2 were further examined.

Following the results of this initial modelling process, separate general linear regression models for each species were constructed and assessed using a similar procedure, where the global model for each species was lnfCort ~ Year + δ^13^C + δ^15^N. Again, only models with ΔAIC_c_ < 2 were used for inference (Burnham and Anderson, 2004). For the top models between species and within species, the amount of variance explained by each model was calculated by dividing the model deviance by the null deviance and subtracting this value from 1 (‘model fit’).

Tukey tests were also performed for each species to assess differences in lnfCort across years, based on results of the model selection (Table 1). Analysis of variance was first conducted using the ‘aov’ function, then the ‘TukeyHSD’ function from the ‘multcompView’ package was used on the resulting model at 95% confidence.

Based on significant interannual variation in lnfCort values in Cassin’s auklets but not in rhinoceros auklets, we explored the relationship between fCort and fmPDO, an index representing fluctuations in the Pacific Decadal Oscillation (http://research.jisao.washington.edu/pdo/PDO.latest). We used averaged PDO values from February and March of each year (fmPDO), representing the pre-breeding period most critical to breeding health (Crossin et al., unpublished work) and when peak feather growth occurs (Pyle, 2008). A general linear model was assessed with fmPDO as the predictor and lnfCort as the dependent variable for each species.

Differences in feather δ^15^N and δ^13^C across years were also explored using the ‘aov’ and ‘TukeyHSD’ function at 95% confidence to further examine trophic preferences throughout the Blob. Each isotope was used as a dependent variable with year as the predictor and tested separately for each species.

## 3. Results

When combined data from Cassin’s and rhinoceros auklets were analysed together, lnfCort varied primarily among species and year (Table 1). We subsequently analysed each species separately to incorporate the feather isotopes into the models and to simplify the analysis. The best supported model for the Cassin’s auklets indicated that lnfCort varied primarily with year (Table 2). For the rhinoceros auklets, the top model was the null model with no predictors (Table 2). Two additional models with ΔAIC_c_ < 2 were also supported for the rhinoceros auklets, the first with year as the only predictor of lnfCort and the second with δ^13^C as the only predictor (Table 2).

**Table 2:** Models predicting lnfCort for female Cassin’s auklets (*P. aleuticus*) and rhinoceros auklets (*C. monocerata*), with separate models run for each species. The global model was lnfCort~ Year + δ^13^C + δ^15^N. ΔAIC_c_ is the difference between a given model and the top-ranked model, AIC_w_ is the Akaike model weight, model fit provides a measure analogous to R^2^, and is calculated by 1—model deviance/null model deviance where a fit closer to 1 is best. Models with ΔAIC_c_ < 2 are in bold

Parameters	AIC_c_	ΔAIC_c_	AIC_w_	Model fit
Cassin’s auklets				
**Year**	**30.3**	**0.00**	**0.561**	**0.53**
δ^13^C + Year	32.5	2.15	0.191	0.54
δ^15^N + Year	32.5	2.19	0.187	0.54
δ^15^N + δ^13^C + Year	34.8	4.45	0.061	0.54
~1 (null model)	107	77.1	0.000	0.00
δ^15^N	109	79.1	0.000	0.00
δ^13^C	110	79.2	0.000	0.00
δ^15^N + δ^13^C	111	81.1	0.000	0.00
Rhinoceros auklets				
**~1 (null model)**	**−8.5**	**0.00**	**0.287**	**0.00**
**Year**	**−7.3**	**1.21**	**0.157**	**0.09**
**δ** ^**13**^**C**	**−7.3**	**1.24**	**0.154**	**0.01**
δ^15^N	−6.5	2.04	0.103	0.00
δ^15^N + δ^13^C + Year	−6.4	2.09	0.101	0.14
δ^15^N + Year	−5.9	2.63	0.077	0.10
δ^13^C + Year	−5.6	2.87	0.068	0.10
δ^15^N + δ^13^C	−5.1	3.41	0.052	0.01

**Figure 1:**
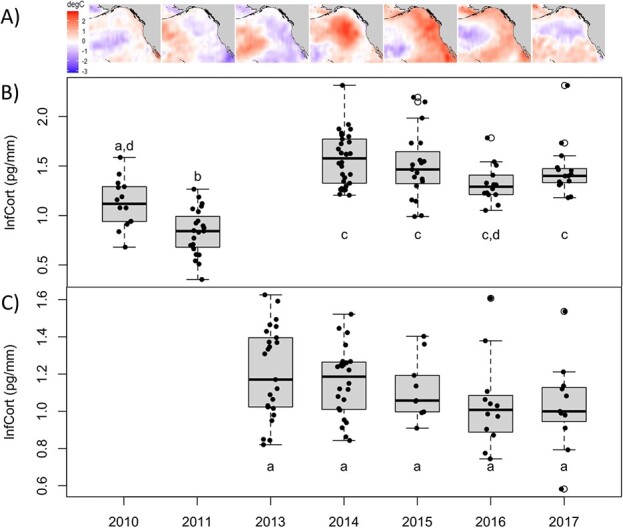
Sea surface temperature anomalies during peak feather growth (February–March) from 2010 to 2011 and 2013 to 2017. Years 2010, 2011 and 2013 occurred before the marine heatwave (‘the Blob’) moved closer to shore, in contrast to other years during and following the Blob, which peaked 2014–2016 (**A**). Temperature data are from NOAA’s NCEP. Corresponding lnfCort (pg/mm) data for female Cassin’s auklets (*P. aleuticus*) and rhinoceros auklets (*C. monocerata*) are presented, respectively, in (**B**) and (**C**). Letters show annual differences from Tukey tests at 95% confidence. For each box, the interior black line represents the median, the box represents the interquartile range, the whiskers show the minimum and maximum without outliers and the circular points represent outliers.

Average lnfCort values of Cassin’s auklets differed across years, being significantly lower in 2010 and 2011 than in 2014–2017 (Fig. 1B). There was, however, no difference between lnfCort in 2010 and 2016. From the AIC_c_ models tested for the rhinoceros auklets (Table 2), the null model had the lowest AICc value, followed by the model that included year, indicating that fCort did not vary strongly among years. Follow-up Tukey tests supported this inference for rhinoceros auklets and indicated there were no interannual differences in lnfCort across years from 2013 to 2017 (Fig. 1C).

**Figure 2:**
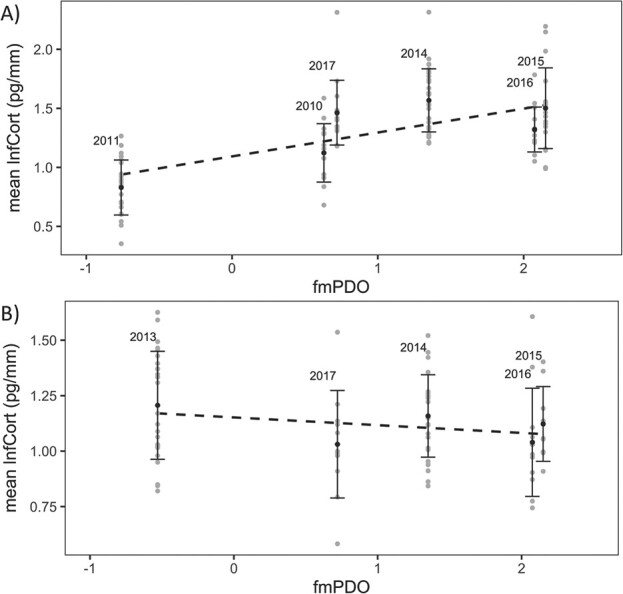
Mean lnfCort (black) and individual lnfCort levels (grey) of female Cassin’s auklets (*P. aleuticus*) (**A**) and Rhinoceros auklets (*C. monocerata*) (**B**) compared to the February–March Pacific Decadal Oscillation index (fmPDO) in the year of sample collection. Dashed lines illustrate a general linear model for Cassin’s auklets (R^2^ = 0.37, intercept = 1.09, slope = 0.22, std. error = 0.03, *P* < 0.0001) and rhinoceros auklets (R^2^ = 0.04, intercept = 1.17, slope = −0.04, std. error = 0.02, *P* = 0.09).

Cassin’s auklet lnfCort levels also showed a positive, linear relationship with fmPDO values (R^2^ = 0.37; Fig. 2A), while lnfCort levels in rhinoceros auklets were not significantly related to the fmPDO index (R^2^ = 0.04 Fig. 2B).

**Figure 3:**
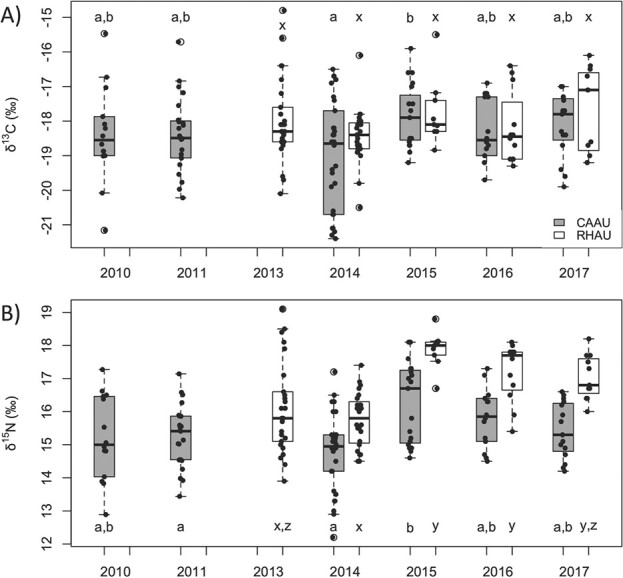
Feather isotope (‰) data for female Cassin’s auklets (*P. aleuticus*; CAAU) and rhinoceros auklets (*C. monocerata*; RHAU) for δ13C, representing potential differences in use of benthic versus pelagic prey (panel A), and δ15N, representing relative trophic levels (panel B). Letters show annual differences from Tukey tests for each separate species and isotope at 95% confidence. For each box, the interior black line represents the median, the box represents the interquartile range, the whiskers show the minimum and maximum without outliers, and the circular points represent outliers.

Tukey tests for feather isotopes showed no difference across most years for δ^13^C in either species, apart from 2014 and 2015 for Cassin’s auklets, suggesting little to no change in foraging area or benthic versus pelagic prey inputs to diets (Fig. 3A). Values of δ^15^N showed no difference across years for the Cassin’s auklets apart from 2014–2015 and 2011–2015, potentially demonstrating some increase in their prey’s trophic level in 2014 and 2015. In contrast, δ^15^N was elevated from 2015–2017 for the rhinoceros auklets, possibly indicating prey from higher trophic levels or increased nutritional stress (Hobson *et al.*, 1993; Fig. 3B). Despite these apparent trends, no relationship was present between feather isotopes and lnfCort for either species (Table 2), so changes in isotopes throughout the Blob were unrelated to measures of fCort.

## 4. Discussion

Using data collected over an 8-year period, we examined the physiological response of female Cassin’s and rhinoceros auklets during the pre-breeding period to an extreme marine heatwave in the Northeast Pacific, known as the Blob (Kintisch, 2015). Here, we reveal interannual differences in fCort of Cassin’s auklets (Fig. 1B; Table 2), which provides a measure of the allostatic load or cumulative stress experienced during the period of feather growth in mid-winter (February–March; Fairhurst *et al.*, 2017; Studholme *et al.*, 2019). Additionally, this interannual variation in fCort was correlated with the strength of a regional index of climate variability, the Pacific Decadal Oscillation from which the Blob developed (Fig. 2A). February is a critical period for female Cassin’s auklets, as variation in productivity and prey distributions at this time can generate carry-over effects that influence breeding phenology months later, such as lay date, egg size and reproductive success (Crossin et al., unpublished work; Sorensen *et al.*, 2009), and these environmental conditions can vary considerably depending on sea surface temperatures (Du and Peterson, 2018; Jones *et al.*, 2018; Piatt *et al.*, 2020). In contrast, female rhinoceros auklets did not exhibit any fCort differences across years (Fig. 1C; Table 2), suggesting greater resilience to climate variation in mid-winter (Fig. 2B). This difference is consistent with the higher and more stable survival rates of rhinoceros auklets compared to Cassin’s auklets observed during previous marine heatwaves (Morrison *et al.*, 2011), as well as a tendency for higher adaptability in larger seabirds like rhinoceros auklets than smaller seabirds like Cassin’s auklets (Sandvik and Einar, 2008).

One of the most conspicuous effects of the Blob was a reduction in phytoplankton abundance (Kintisch, 2015; Lotze *et al.*, 2019; Suryan *et al.*, 2021) and a decrease in cold-water copepods with a northward shift of warm-water copepods within the migratory range of both auk species (Hipfner *et al.*, 2020b; Jones *et al.*, 2018; Studholme *et al.*, 2019). For Cassin’s auklets, which rely heavily on specific cold-water copepod species (e.g. sub-Arctic *Neocalanus cristatus;*  Hipfner *et al.*, 2020a), this likely caused a disruption to their foraging ecology and increased nutritional stress (Jones *et al.*, 2018). This idea is corroborated by fCort values, which were lower during the cooler, negative phase of the PDO in 2010 and 2011 prior to the start of the heatwave and higher during the positive Blob phase (Fig. 2A). Lowered observed fCort values in cooler years was especially obvious in 2011, when the PDO was at its most negative phase, likely allowing for increased primary productivity and available food sources (Du and Peterson, 2018; Fig. 1A). As well, fCort was higher than expected in 2017 based on the lowered PDO value that year (Fig. 2A), potentially related to lagging ecosystem recovery after a marine heatwave (Suryan *et al.*, 2021).

Although feather isotopes differed across some years (Fig. 3), they were not strong predictors of fCort (Table 2), so any changes in diet reflected in the feather isotopes were unrelated to nutritional stress. Throughout the Blob, some groups of Cassin’s auklets had modified their migratory ranges slightly (Studholme *et al.*, 2019), which may explain small changes in δ^13^C values across some years, although it is unlikely this had a significant effect on physiology as the Blob was far-reaching. Feather δ^15^N was also slightly elevated throughout 2015–2017, which may be indicative of feeding at slightly higher trophic levels, although this change was not as pronounced as for the rhinoceros auklets. That neither isotope was a strong predictor of fCort in the Cassin’s auklets suggests a lower adaptability and potentially less success at maintaining regular fCort levels. The fCort levels recorded in this study were from surviving individuals, so increased fCort throughout the Blob may have promoted increased foraging effort as prey abundance decreased (Landys *et al.*, 2006; Wingfield *et al.*, 1998). Future research should examine links between mid-winter climate and fCort as a mediator of carryover effects on reproductive processes. For example, previous studies of other seabird species have demonstrated links between decreased body condition and reproductive success with increased fCort (Fairhurst *et al.*, 2017; Harms *et al.*, 2014). Isotopic and fCort sampling of birds that do not survive such heatwaves (e.g. from seabird mass die-offs) compared to those that do would also be informative.

In contrast, fCort levels in female rhinoceros auklets were largely unchanged from mid-winter 2013, when the Blob was forming offshore but had not yet overlapped their migratory range, to 2014–2016, when the Blob had entirely overlapped their range and after the Blob’s decline in 2017 (Hipfner *et al.*, 2020b; Kintisch, 2015; Fig. 1B). This temporal pattern in rhinoceros auklets suggests greater resiliency against nutritional stress than Cassin’s auklet, possibly related to this species’ broader diet which includes both zooplankton and fish, which could buffer against reductions in overall prey biomass in warm years (Carle *et al.*, 2015; Hipfner *et al.*, 2013). Changes in feather isotope values and diet were unrelated to nutritional stress as any annual variations in fCort were insignificant for the rhinoceros auklets (Table 2) (Hobson *et al.*, 1993). However, feather δ^15^N was higher during the Blob in 2015 and 2016, and throughout recovery in 2017 (Fig. 3B), an indication of possible higher trophic-level prey (Hipfner *et al.*, 2014). As zooplankton populations decrease and forage fish populations shift in distribution throughout marine heatwaves (Cavole *et al.*, 2016), stable fCort values and variable isotopes across the Blob demonstrate the advantages of generalist feeding in rhinoceros auklets (Carle *et al.*, 2015). Our results therefore indicate higher adaptability to the Blob in rhinoceros auklets than in Cassin’s auklets. However, we note that data were collected in only one cold-water year for the rhinoceros auklets (in 2013) from which no Cassin’s auklet samples were available and that the pre-Blob years sampled for each species were different, so we cannot rule out the possibility of across-year effects having had an impact on our conclusions drawn about differences across species. Future studies should confirm these findings with measurements from the same additional cold years from both species for comparison.

As the global climate continues to warm, both the frequency and intensity of marine heatwaves are expected to increase (Joh and Di Lorenzo, 2017; Oliver *et al.*, 2018). Increased nutritional stress, die-offs and carryover effects onto breeding parameters are likely consequences for North Pacific seabirds including Cassin’s auklets, as recovery time is decreased between heatwaves (Suryan *et al.*, 2021). Results of our study suggest that monitoring fCort levels in seabirds could reveal impacts of climate change on marine ecosystem health in the Northeast Pacific. In just the past decade, various record-breaking ocean-warming events have occurred, including the ‘Ningaloo Niño’ off Western Australia (Pearce and Feng, 2013) and the extreme El Niño that affected most of the Indo-Pacific in 2016 (Benthuysen *et al.*, 2018). Elsewhere, marine heatwave-related decreases in primary productivity have also been correlated with decreased survival and breeding success of the following: Atlantic puffins (*Fratercula arctica*), common terns (*Stirna hirundo*) and Cory’s shearwater (*Calonectris diomedea*) in the Atlantic (Jenouvrier *et al.*, 2009; Morley *et al.*, 2016; Szostek and Becker, 2015); king penguins (*Aptenodytes patagonicus*) in the Southern Ocean (Le Bohec *et al.*, 2008); and roseate terns (*Sterna dougallii*) in the western Indian Ocean (Monticelli *et al.*, 2007). Other marine vertebrates including marine mammals and fish have also been negatively affected by such phenomena due to bottom-up effects of ecosystem shifts (Sydeman *et al.*, 2015). As studies suggest, marine heatwaves will amplify these effects in the future, causing potentially irreversible changes to ecosystem health (Laufkötter *et al.*, 2020; Sydeman *et al.*, 2015).

## Funding

Major funding for this research was provided by Environment and Climate Change Canada's A-base and Oceans Protection Plan funded to J.M.H., the National Sciences and Engineering Research Council of Canada's Discovery grants [nos. 04044-2014 and 4374-2015 RGPIN] funded to G.T.C., and K.R.S.’s Graduate Student Research Award from the North Pacific Research Board (2014). fCort analyses were funded by U.S. National Science Foundation grant IOS-1655269 to L.M.R. B.M.G. is currently funded by a fellowship through the Grand Challenges Initiative at Chapman University.

## Author’s contributions

J.M.H., M.C.D., K.R.S., A.D.D. and G.T.C. collected and compiled data from Triangle Island. L.M.R. and B.M.G.G. performed corticosterone analysis. K.A.H. performed stable isotope analysis. H.M.T. performed statistical analyses and wrote the manuscript. All co-authors provided input to the final manuscript.

## A. Appendix

### Appendix

**Table A1:** Annual feather corticosterone (pg/mm) mean, standard deviation, and sample number for female Cassin’s auklets (*Ptychoramphus aleuticus*) and rhinoceros auklets (*Cerorhinca monocerata*)

Year	Mean	Standard deviation	N
Cassin’s auklet			
2010	3.16	0.79	14
2011	2.35	0.55	23
2014	4.97	1.46	30
2015	4.76	1.79	19
2016	3.81	0.80	14
2017	4.51	1.66	15
Rhinoceros auklets			
2013	3.44	0.83	25
2014	3.24	0.60	24
2015	3.11	0.55	9
2016	2.91	0.81	12
2017	2.88	0.73	11

**Table A2:** Annual feather isotope (‰) mean, standard deviation, and sample number of δ^15^N and δ^13^C for female Cassin’s auklets (*Ptychoramphus aleuticus*) and rhinoceros auklets (*Cerorhinca monocerata*)

Year	Mean (‰)		Standard deviation (‰)		N
Cassin’s auklet	δ^15^N	δ^13^C	δ^15^N	δ^13^C	
2010	15.22	−18.39	1.29	1.40	14
2011	15.24	−18.41	0.94	1.08	23
2014	14.80	−19.02	1.17	1.61	30
2015	16.26	−17.85	1.23	0.93	19
2016	15.81	−18.28	0.90	0.92	14
2017	15.43	−18.13	0.85	0.94	15
Rhinoceros auklets	δ^15^N	δ^13^C	δ^15^N	δ^13^C	
2013	16.12	−17.98	1.39	1.23	25
2014	15.77	−18.43	0.82	0.80	24
2015	17.88	−17.74	0.57	0.98	9
2016	17.22	−18.21	0.89	1.04	12
2017	17.04	−17.69	0.68	1.24	11
